# Raising the bar in respiratory care by EUFOREA: report of the European Union Parliament Symposium, April 2024

**DOI:** 10.3389/falgy.2025.1540499

**Published:** 2025-02-24

**Authors:** P. W. Hellings, D. M. Conti, X. Bertels, V. Backer, G. Brusselle, E. De Corso, W. J. Fokkens, A. T. Fox, P. Gevaert, S. Lau, G. Liva, S. Palkonen, A. Papi, S. Scheire, P. Schmid-Grendelmeier, C. M. E. Shire, P. Smith, M. T. A. Teeling, A. Yorgancioglu, G. K. Scadding

**Affiliations:** ^1^ Department of Otorhinolaryngology, Laboratory of Upper Airways Research, University of Ghent, Ghent, Belgium; ^2^ Department of Microbiology and Immunology, Allergy and Clinical Immunology Research Unit, KU Leuven, Leuven, Belgium; ^3^ Clinical Department of Otorhinolaryngology, Head and Neck Surgery, University Hospitals Leuven, Leuven, Belgium; ^4^ Escuela de Doctorado UAM, Centro de Estudios de Posgrado, Universidad Autónoma de Madrid, Madrid, Spain; ^5^ Allergy and Clinical Immunology Research Unit, Department of Microbiology and Immunology, KU Leuven, Leuven, Belgium; ^6^ Scientific Expert Team Members, The European Forum for Research and Education in Allergy and Airway Diseases, Brussels, Belgium; ^7^ Department of Epidemiology, Erasmus MC, Rotterdam, Netherlands; ^8^ Department of Otorhinolaryngology, Head & Neck Surgery, and Audiology, Rigshospitalet, Copenhagen University, Copenhagen, Denmark; ^9^ Department of Respiratory Medicine, Ghent University Hospital, Ghent, Belgium; ^10^ Departments of Epidemiology and Respiratory Medicine, Erasmus Medical Center Rotterdam, Rotterdam, Netherlands; ^11^ Otorhinolaryngology Unit, A. Gemelli University Hospital Foundation IRCCS, Rome, Italy; ^12^ Department of Otorhinolaryngology, Amsterdam University Medical Centre, Amsterdam, Netherlands; ^13^ Children’s Allergy Service, Evelina London Children’s Hospital, London, United Kingdom; ^14^ Department of Paediatric Allergy, King’s College London, London, United Kingdom; ^15^ Upper Airways Research Laboratory, Department of Head and Skin, Ghent University, Ghent, Belgium; ^16^ Department of Pediatric Respiratory Medicine, Immunology and Critical Care Medicine, Charité Universitätsmedizin Berlin, Berlin, Germany; ^17^ Department of Otorhinolaryngology, School of Medicine, University of Crete, Heraklion, Greece; ^18^ European Federation of Allergy and Airways Diseases Patients’ Associations (EFA), Brussels, Belgium; ^19^ Respiratory Medicine, Univerity of Ferrara, Ferrara, Italy; ^20^ Allergy Unit, Department of Dermatology, University Hospital of Zurich, Zurich, Switzerland; ^21^ Department of Dermatology, High Altitude Clinic, Medicine Campus Davos-Wolfgang, Davos-Wolfgang, Switzerland; ^22^ Patient Advisory Board, The European Forum for Research and Education in Allergy and Airway Diseases, Brussels, Belgium; ^23^ School of Medicine, Griffith University, Gold Coast, QLD, Australia; ^24^ Menzies Centre, National Centre for NeuroImmunology and Emerging Disease, Gold Coast, QLD, Australia; ^25^ Department of Chest Diseases, Celal Bayar University Faculty of Medicine, Manisa, Türkiye; ^26^ Department of Allergy & Rhinology, Royal National ENT Hospital, London, United Kingdom; ^27^ Division of Immunity and Infection, University College, London, United Kingdom

**Keywords:** EUFOREA, European Parliament, respiratory care, action plan, chronic respiratory diseases

## Abstract

In April 2024, the European Summit “*Raising the bar in respiratory care”* was organized by the European Forum for Research and Education in Allergy and Airway Diseases (EUFOREA) in the European Parliament and hosted by Members of the European Parliament Dorien Rookmaker and Mislav Kolakušić. The aim of the Summit was to respond to the need of European patients suffering from chronic respiratory diseases (CRDs) by joining forces with European and global organisations in the management of the epidemics of CRD, recognising the weaknesses of current care models and focussing on collaboration to improve care and prevention. Participants belonging to International and National Societies and Committees from the European Rhinologic Society (ERS), International Rhinologic Society (IRS), Belgian Respiratory Society (BeRS), Global Initiative for Asthma (GINA), Global Initiative for Chronic Obstructive Lung Disease (GOLD), Global Alliance against Chronic Respiratory Diseases (GARD), and from the European Federation of Allergy and Airways Diseases Patients Associations (EFA) and the EUFOREA's Patient Advisory Board (PAB) described their vision and action plan to work in partnership to raise the bar in respiratory care. This report summarizes the contributions of the representatives of different European stakeholders in the field of CRDs.

## Introduction

The World Health Organization (WHO) rates CRDs as one of the 4 major chronic diseases of mankind (1). CRDs often start in early childhood and persist throughout the life cycle. They have had a significant impact on society at large and are a major cause of economic burden (2). CRDs represent a major global health problem leading to gender and social inequalities within and between countries (3). CRDs including asthma, chronic obstructive pulmonary disease (COPD), allergies and chronic rhinosinusitis (CRS) are complex diseases intertwined with ageing. Asthma, CRS and allergic rhinitis (AR) are the most common non-communicable diseases in children and adults, and their prevalence and burden have increased in recent decades, reaching epidemic proportions (4–8).

In 2021, the European Commission launched “Healthier together – EU non-communicable diseases (NCD) initiative” (9) to support EU countries in identifying and implementing effective policies and actions to reduce the burden of major non-communicable diseases, and to improve citizens' health and well-being. CRDs were one of five focus areas along with cardiovascular diseases, diabetes, mental health and neurological disorders, and health determinants. Each focus area aims to address health inequity as well as to support improved knowledge and data, diagnosis and treatment, management and the improvement of patients' quality of life. These EU-wide ambitions are at the heart of EUFOREA's mission of “*Raising the bar in Respiratory Care*.” EUFOREA takes a holistic approach in its programme development- including representation by patients in discussions with medical experts and health policy makers (10–12) and including those organisations that are open to join forces to tackle the current weaknesses of respiratory care, i.e., lack of prevention, lack of value- based health care, lack of patient empowerment and lack of a multidisciplinary approach considering the respiratory tract as a single entity. In addition to the repeated political advocacy activities of EUFOREA in the EU Parliament since 2015 (2, 13), in which European patients and experts have highlighted the major needs of citizens and patients, EUFOREA aims to optimize respiratory care in daily practice via research, educational and advocacy.

**Figure 1:**
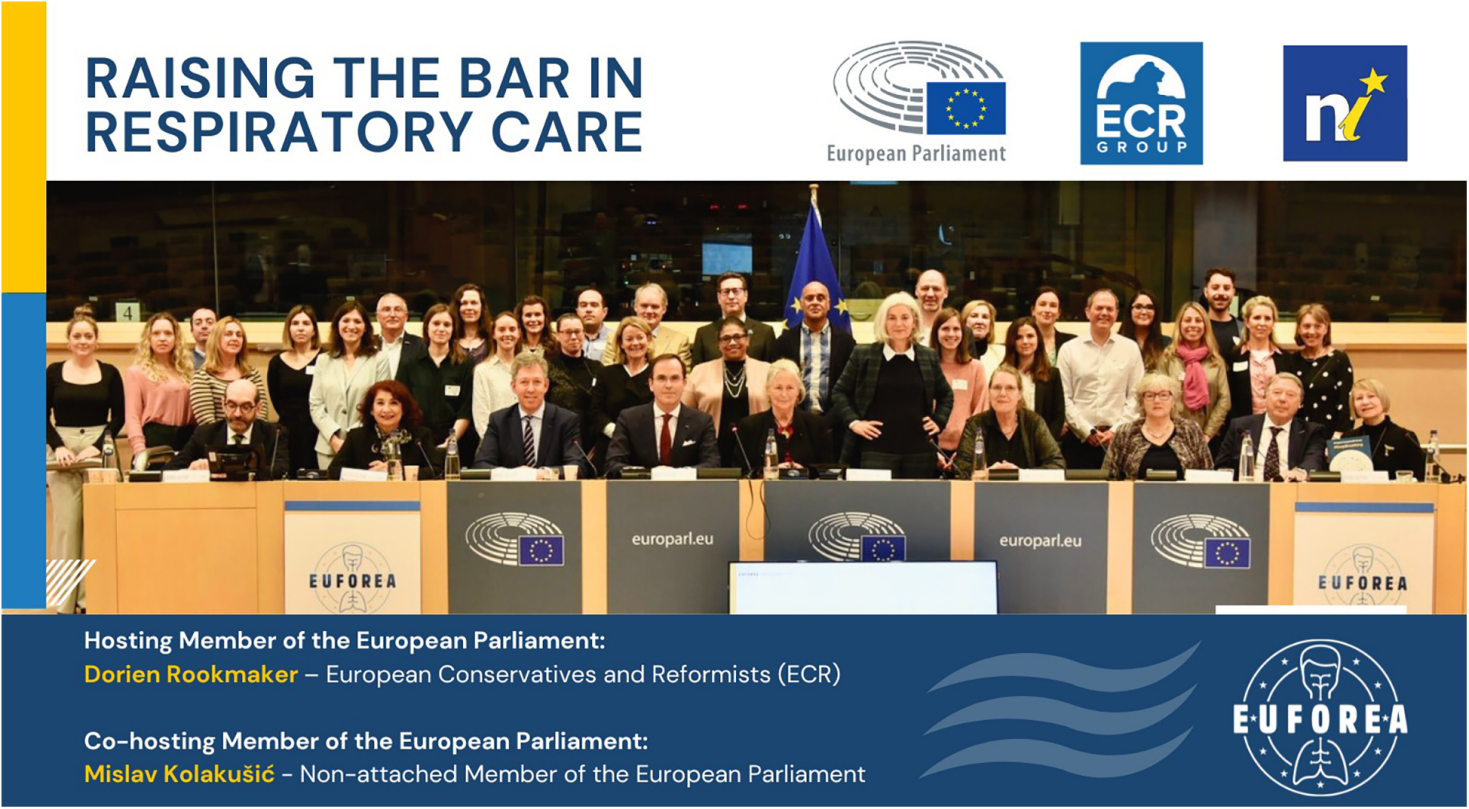
European Union Parliament Symposium group photo.

This report summarizes the contributions of major organisations dealing with the preventable burden of CRDs (Figure 1).

### The vision, mission and objectives of EUFOREA

EUFOREA is an international non-for-profit organization forming an alliance of major stakeholders from national and international organizations, institutions, and agencies working towards the common target to implement optimal care for CRDs and comorbidities. EUFOREA aims to reduce the preventable and avoidable burden of morbidity and disability due to CRDs by means of a multidisciplinary practical approach, engaging the best specialists within the upper and lower airways domains. Its aim is to prevent CRDs from being a barrier to well-being and socio-economic development, enabling populations to reach the highest attainable standards of health and productivity at every age. Given that CRDs represent a continuum of pathophysiological processes (4–7), and that an untreated or inadequately- treated patient is expected to present with more severe stages of their disease and associated comorbidities (4–7), the cost of inaction is unacceptable and integrated action is urgently needed in Europe and beyond. Such action must be multi-disciplinary, involving all relevant stakeholders.

The main objective of EUFOREA is to initiate a comprehensive approach to prevent and fight CRDs in upper and lower airways via education of physicians and patients alike, research on unmet needs, and advocacy.

• To better educate patients, the (para)medical community and the public, allowing them to be empowered for prevention, self-management and optimal care in the early stages of disease
• To better educate health care providers on the optimal care pathways for CRDs from a holistic perspective, taking into account the whole respiratory tract and the multidisciplinary nature of the co-morbidities of those suffering from asthma, COPD, CRS and AR
• To promote active and healthy lifestyles and healthy ageing
• To improve the well-being of people suffering from chronic airway disease
• To improve the work productivity of people suffering from chronic airway disease
• To reduce health and social inequities
• To amplify the voices of patients with CRDs and recognize their needs, as well as the impact of the disease on their daily lives

The value of EUFOREA lies in its ability to develop a strategic partnership for the prevention and control of CRDs. This partnership addresses all aspects of prevention, from the basic science underlying the disease to the policies that regulate its impact. EUFOREA brings together specialists from both primary and secondary care, as well as public and private healthcare (4–7), and has established an international patient advisory board (PAB) to ensure the pro-active participation of healthcare users in debates, consensus meetings and advocacy events (10–12). EUFOREA facilitates the generation of novel, innovative approaches to care and the development of optimal care strategies. EUFOREA produces pocket guides for primary and secondary care professionals, within our areas of expertise, for CRDs such as asthma, CRS and allergy for both children and adults; and also, web-based e-learning platforms, podcasts, face-to-face scientific meetings and masterclasses. In addition, it attempts to improve coordination between existing EU, governmental and non-governmental programmes to avoid duplication of efforts and wasting of resources.

### Perspectives on shortcomings of respiratory health

The implementation of a global programme must be based on a solid foundation. In order to achieve this objective, it is of the utmost importance to gain an understanding of the limitations in respiratory health. Currently, in the EU, more than 10 million individuals with mild asthma lack access to the preferred treatment: a combination of inhaled corticosteroid (ICS) and formoterol as anti-inflammatory reliever in these patients which has been supported by substantial evidence of efficacy, safety, and favourable benefit/risk (14, 15). As-needed use of ICS/formoterol in mild asthma has been approved in over 50 countries worldwide and is strongly recommended by major guidelines at the European (16) and global (17) levels. Indeed, the European Respiratory Society short guidelines for the use of as-needed ICS/formoterol in mild asthma suggests that adults with mild asthma use as-needed ICS/formoterol instead of regular ICS maintenance treatment plus as-needed short-acting β2-agonist (SABA) and that adolescents with mild asthma use either as-needed ICS/formoterol or ICS maintenance treatment plus as-needed SABA (16). Nevertheless, despite the aforementioned evidence, the drug has not (yet) been approved by the European Medicines Agency (EMA) as anti-inflammatory reliever-only treatment in adults and adolescents with mild asthma. The implementation of such a measure would have a profound and far-reaching impact, not only in terms of a reduction in the rate of asthma attacks but also a reduction in the progression to more severe forms of the disease, including the worsening of the underlying condition and the development of oral corticosteroid induced comorbidities (14–18). It is estimated that 43 million people in the EU currently suffer from asthma, with 17,000 deaths per year attributed to the condition (18). The use of ICS + formoterol in patients with mild asthma has been demonstrated to result in a vast reduction in the frequency of exacerbations and in the likelihood of progression to more severe disease (19).

At the severe end of the asthma spectrum, EUFOREA wants to highlight the significant shortcoming regarding the availability of monoclonal antibodies for the treatment of severe type 2 asthma in many countries. Another unmet need is the necessity for a European platform to facilitate international pragmatic trials to optimize the treatment of asthma and other respiratory diseases following marketing authorization. There is a significant discrepancy between the stages of drug development and the clinical practice of medicine. In the former, studies focus on the efficacy and safety of a drug in strictly selected populations. In contrast, in the latter the drug is used in a more heterogeneous setting, including in multimorbid patients who are generally excluded from traditional clinical trials (20, 21). There are numerous examples of drugs that have been approved for human use, yet there is currently insufficient evidence to determine which drug is most appropriate for which patient, which biomarkers can predict therapeutic response, or how long each patient should be treated. These are key questions that require further investigation. The approach advocated by this group is not novel and is already being implemented in cancer patients. Therefore, we believe it has the potential to become a reality for CRDs as well.

From the patient's perspective, the challenges are of equal importance. Disease progression affects the quality of life of patients on a daily basis, often due to the implementation of inappropriate or delayed treatments. It is still possible to encounter asthmatic patients who present at the hospital for the first time with their first exacerbation, having been afflicted but undiagnosed for some time prior to this. Such circumstances can have a detrimental impact on an individual's physical and mental well-being, potentially leading to feelings of anxiety, a reluctance to engage in physical activity, and a sense of social isolation. In light of these considerations, the formation of multidisciplinary teams that approach the consultation with a comprehensive understanding of the disease's various symptoms becomes increasingly crucial.

The patient is expected to be adequately prepared for the medical consultation. Due to the socio-health context, the allocated time for each patient is reduced, which has a detrimental impact on the quality of medical care. An informed patient is an empowered patient and is therefore better able to make the most of the encounter. Three fundamental questions can be applied to any medical specialty, and their efficacy has been demonstrated to be significant:

• What are my options?
• What are the possible benefits and risks derived from these options?
• What does that mean in my particular situation?
• What is the risk of doing nothing?

It is important for patients to be aware that they are not alone. There are numerous patient or scientific organizations that offer support and educational resources. It is of benefit to patients to be aware of these organizations and to be able to contact them should the need arise. In this context, it is expected that clinicians will adopt a holistic approach. This necessitates the utilization of transparent and comprehensive communication (22), which delineates the procedures to be undertaken in both the short, medium and long term. Furthermore, methodology employed to assess an individual's susceptibility for a particular disease is evolving. It has been demonstrated that the patient is not merely a carrier of a disease, but that they possess unique characteristics that influence its progression. It is the responsibility of healthcare providers to address both issues for the benefit of the patient. EUFOREA has developed and will continue to develop step-by-step guides for each of the airway diseases, which are then adapted to language suitable for patients (4–7).

Nasal inflammatory diseases are highly prevalent. Conditions like AR and CRS reduce the quality of life of those affected, and when not appropriately addressed, may result in lower airways diseases, such as asthma. A clear example is AR, which is frequently underestimated as “just another cold” and inadequately treated. In a clinical-academic endeavour to raise awareness and alert all levels of the health system, EUFOREA has coined the concept of pre-asthma (23, 24). In the case of AR and other conditions, the efficacy of allergen-specific immunotherapy (AIT) has been demonstrated over time. This serves as a clear illustration of the potential impact of a seemingly straightforward and accessible measure in halting the progression of a disease when implemented in an appropriate patient at an early age (25). This group considers that the initial proposal (the wide distribution of EUFOREA guides, patient education, and the increased use of AIT in childhood allergic rhinitis) represents a significant step forward and will have a profound impact on the lives of many patients. A plethora of international guidelines are available that support and address this point; however, the EUFOREA AR expert consensus guidelines adopt a multidisciplinary approach, considering all links in the health system chain and provide a clear initial pathway using AIT for those desiring cessation of disease progression. Dissemination of these guidelines is proceeding apace with translations into several languages. In addition, the EUFOREA Patient Portal will be expanded shortly to include AR. Patients will be able to discover the benefits of AIT and be encouraged to request consideration of its use for themselves.

## The role of scientific and patient societies

CRDs have reached epidemic proportions (26). In addition to the exponential increase in prevalence over the past 3 decades, there has been a proportional rise in mortality rates (27). The mortality figures within the group are led by asthma, COPD and interstitial lung disease (26, 27). Initiatives such as the Lancet Commission are designed to alleviate this situation. The central objective of the initiative is to eliminate COPD. As part of the proposed measures, it seeks to reduce the levels of exposure to smoke, smog, industrial waste in the air and others, recognizing exposure as one of the triggering or worsening factors (28). In tandem with risk factor mitigation and primary prevention, new tools and treatments need to be developed to improve disease control in patients with established disease (29). Spirometry is a cost-effective and readily accessible diagnostic method that can be utilized by all levels of the healthcare system (30). It is imperative that the use of such well-established tool is promoted and facilitated in clinical practice. Missing the opportunity to diagnose COPD is avoidable by simple training in making airway measurements. To this end, additional efforts in raising awareness and aiding primary care to enable its efficient implementation are urgently needed. Also, COPD is rarely diagnosed in isolation; rather, it is frequently accompanied by other comorbidities (31). Therefore, it is crucial to suspect, diagnose, assess and treat these early on to improve the overall prognosis. A multidisciplinary approach must be employed in order to address this problem in a comprehensive manner. In light of the current availability of digital tools such as telemedicine, it is perplexing why this technology is not more widely employed for the benefit of the patient. This could play a primary role in the education, consultation, medical visit and rehabilitation of the patient who requires it. However, digital tools must meet certain criteria to be appealing for patients as well as for physicians (32). Whilst it is acknowledged that there are already established tools which have been shown to be effective (33, 34), it is frequently the case that patient adherence to these tools is low. It is imperative that greater efforts are made and more effective strategies are employed to promote these tools and their benefits among healthcare providers and amongst patients if they are to be fully utilized. EUFOREA is embarking on an online Patient Portal to aid education of patients in this respect and in many other facets of their diseases.

Two illustrative examples of the manner in which organizations can positively influence patients' illness journeys are provided by GARD and EFA. GARD is notable for its connection with the WHO and the inclusion of a collaborative approach, in which professionals, medical societies, patients' organizations, medical and pharmaceutical companies, NGOs, and governments work together towards a common objective. EFA is represented in more than 26 countries and has a vision focused on the best care for all patients in the EU. Its objective is to create a better environment and better decision-making processes for the development of therapeutic approaches. Both have demonstrated the efficacy of inclusive policies with far-reaching impact. An illustrative example is the 2030 Agenda for Sustainable Development, which encompasses the following objectives: a 30% reduction in premature mortality from NCDs, the promotion of mental health and well-being through prevention and treatment, the achievement of universal health coverage, including financial risk protection, the assurance of quality essential health-care services, and access to safe, effective, quality and affordable essential medicines and vaccines for all individuals. This ambitious approach is based on three priority areas: The objective is to enhance access to effective care for individuals with asthma and COPD, to reduce exposure to key risk factors, including tobacco and air pollution, and to expand and strengthen their network.

Many patients with lower airway disease also have upper airways problems which have a significant impact on their quality of life. Allergic rhinitis is present in 20%–50% of the European population, while chronic rhinosinusitis (CRS) affects 5% and asthma 8.5% (35–37). The financial implications of CRDs must be taken into account. With regard to indirect costs, it is estimated that the annual costs of rhinitis in Europe range from 0.5 to 1.2 billion euros per million inhabitants. The direct costs of CRSwNP have been estimated at €1,501 per patient per year, while the costs of asthma have been set at 0.1–0.2 billion euros per inhabitants per year (35–39). Furthermore, indirect costs associated with work absenteeism, relapses, illness, disease progression, and the presence of co-morbidities must be considered. These additional costs result in a significant increase in the overall financial burden (39–41).

It is imperative that medical practitioners receive adequate training to ensure that they are able to provide accurate and up-to-date diagnoses and treatments. ERS is dedicated to the advancement of research through the implementation of educational programmes and events. Its role in the development of international guidelines such as EPOS 2020 (42) has demonstrated significant benefits, including a reduction in the overuse of antibiotics. These examples illustrate the significant impact that well-designed measures can have.

While the current therapeutic goal for CRS patients is control, we strive for remission or disease and eventual cure of the disease and its airway comorbidities (43–48).

### Action plan to join forces with all stakeholders in respiratory care

In light of the socio-health context, it is necessary to implement a comprehensive action plan. It is insufficient merely to engage all levels of the health system; rather, a comprehensive plan for patient education, awareness-raising and empowerment is required. Health systems will be unable to address this issue effectively if they act in isolation. Therefore, a global effort is required.

**Figure 2:**
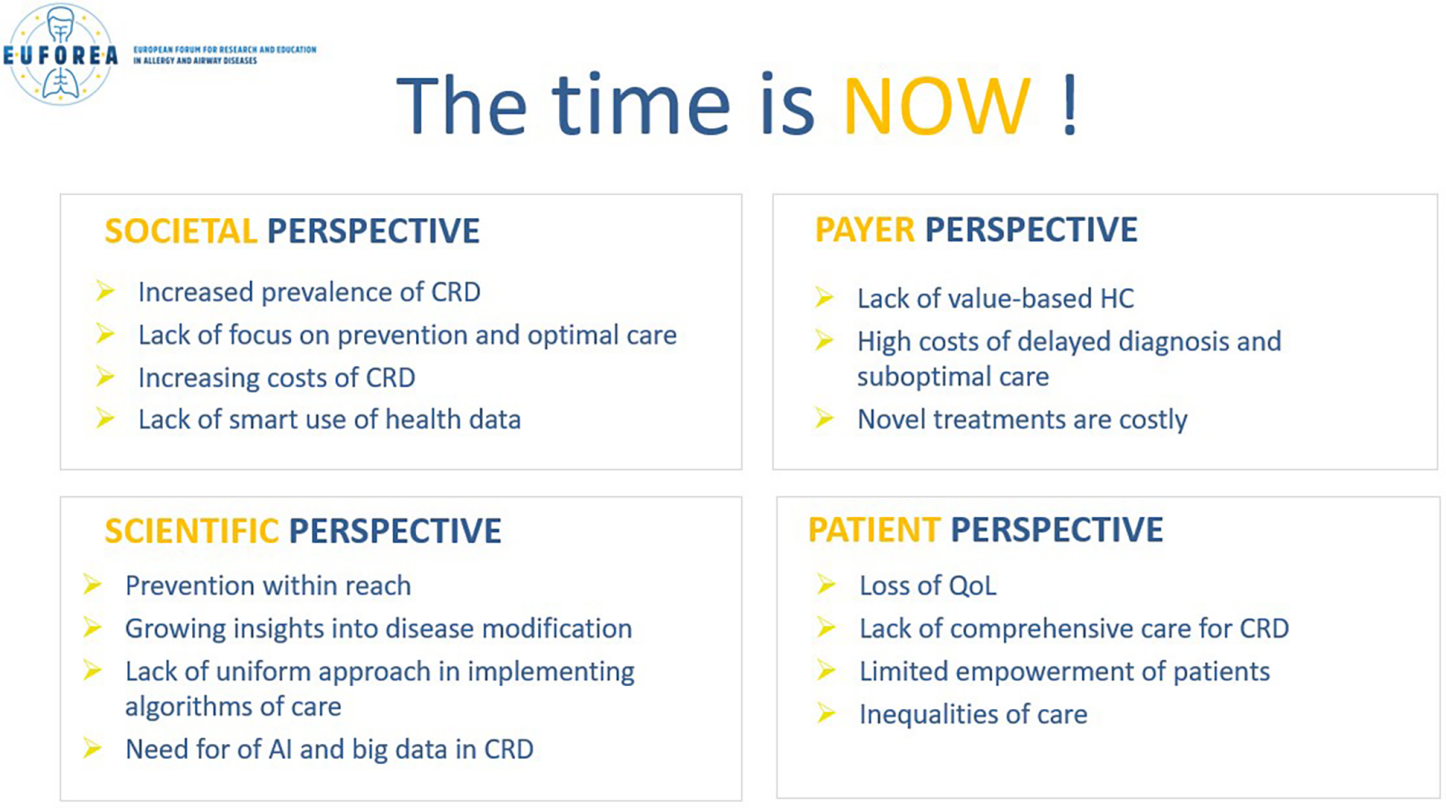
The time is now!

Deficiencies are evident not only from a societal perspective, but also from a payer, scientific and patient perspective (Figure 2). The prevalence of CRDs have increased, and there has been a lack of focus on prevention and optimal care. The costs associated with CRDs and their diagnosis and suboptimal care have increased. There are growing insights into disease modification, however novel treatments are increasingly expensive. The absence of a unified and explicit approach to implementing existing care algorithms has resulted in a decline in the quality of life of patients and an inequitable distribution of care. The costs associated with under-diagnosis, inappropriate choice of treatment, inadequate training of professionals, fall on the patient, who bears the consequences in terms of their health, while the health system must also utilize additional funds and reserves to address the rising costs.

**Figure 3:**
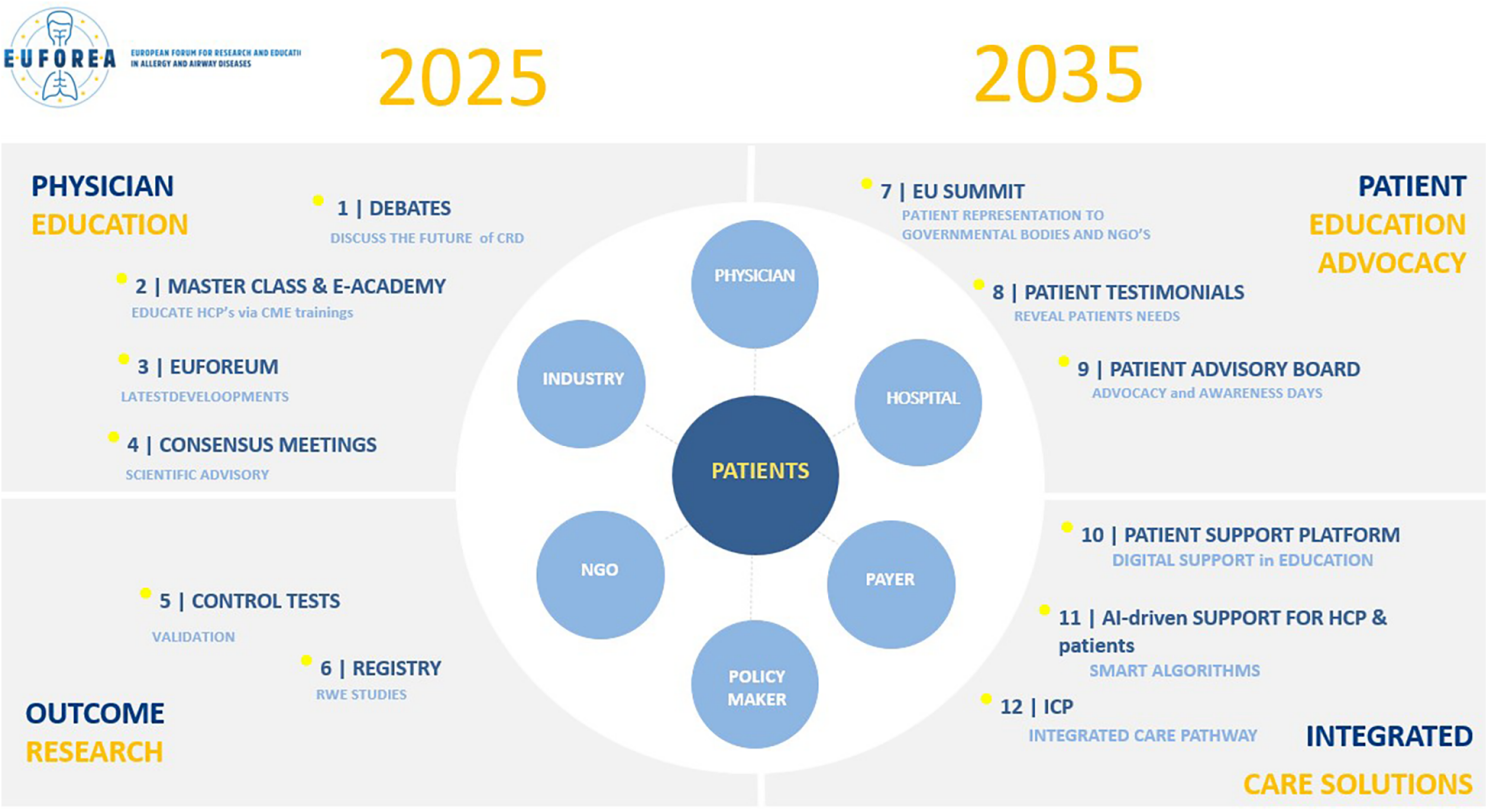
Action plan 2025–2035.

EUFOREA will address this challenge through a global strategy, encompassing four essential areas: physician education, outcome research, patient education advocacy and integrated care solutions (Figure 3). EUFOREA will reinforce its role as an educator by presenting debates and masterclasses, bi-annual editions of the EUFOREUM (49), and consensus meetings on major unmet needs such as definitions of disease states, ambitions of care, screening tools and referral guidance (48). The primary focus of research will be on initiatives related to the creation and validation of new diagnostic tools and real-world efficacy registries at the European level. Patients will assume an increasingly decisive role, continuing to participate in summits (46), providing their testimonies and experiences, and guiding the EUFOREA agenda through their activities at the PAB (10–12).

In terms of integrated solutions, the flagship project will be the Airways Disease Action Plan for Personalised and Preventive Treatment (ADAP^3^T). This will be visible in the form of a patient portal, which will provide patients with reliable information from verified sources such as internationally accepted guidelines, scientific societies and global experts. The programme will commence with a phase dedicated to patient empowerment, followed by a second phase in which patients will be provided with support tools through AI-driven optimal care. Finally, the third phase will demonstrate the impact of the CRDs action plan. Patient education represents the initial stage in the process of empowering patients, which in turn facilitates the reduction of the disease journey and the delivery of the most appropriate treatment to the individual patient. This will only be possible by unifying efforts in a global initiative that adheres to four core values: inclusivity, integrity, responsible use of big data and AI, and unique partnerships.

The European Summit *Raising the bar in respiratory care* demonstrates that the future is bright and this may be indicative of the potential outcomes that can be achieved through a multidisciplinary approach and by collaboration towards a shared common goal.

## Summary

The high prevalence and major socio-economic impact of CRDs require an inter-academic and multi-stakeholder approach for the successful implementation of prevention strategies leading to a reduction of disease burden and cost savings. EUFOREA will continue its mission to implement optimal care to arrest the epidemic of CRD. In Europe, there is an urgent need to work in partnership in the education of patients and medical care providers on prevention strategies, accurate early diagnosis of CRDs, and optimal multidisciplinary treatment of CRDs, and to call for political action supported by all European academic stakeholders involved in the care of CRDs.

## Data Availability

The original contributions presented in the study are included in the article/Supplementary Material, further inquiries can be directed to the corresponding author.
